# Reticuloendothelial system blockade does not enhance siRNA-LNP circulation or tumor accumulation in mice

**DOI:** 10.1016/j.ijpx.2025.100324

**Published:** 2025-02-27

**Authors:** Pol Escudé Martinez de Castilla, Mariona Estapé Senti, Sigrun Erkens, Wytske M. van Weerden, Sander A.A. Kooijmans, Marcel H. Fens, Pieter Vader, Raymond M. Schiffelers

**Affiliations:** a CDL Research, University Medical Center Utrecht, Utrecht, the Netherlands; b Department of Urology, Erasmus MC, Rotterdam, the Netherlands; c Department of Pharmaceutics, Utrecht Institute for Pharmaceutical Sciences (UIPS), Utrecht University, the Netherlands; d Department of Experimental Cardiology, University Medical Center Utrecht, Utrecht, the Netherlands

**Keywords:** siRNA-lipid nanoparticles, Reticuloendothelial system (RES), Tissue distribution, Pharmacokinetics, Liposomes, Dextran sulfate

## Abstract

One of the biggest challenges for siRNA-based therapeutics is intracellular delivery into the target cell, which can be facilitated by encapsulating siRNA in lipid nanoparticles (LNPs). In this study, we formulated D-Lin-MC3-DMA-LNPs encapsulating siRNA against the androgen receptor (AR), a key driver in prostate cancer. We effectively knocked down AR expression at both the mRNA as well as protein levels *in vitro* in AR-expressing prostate cancer cell lines. However, when moving to *in vivo* studies, siRNA-LNP efficacy is hindered by rapid clearance by the reticuloendothelial system (RES) in the liver and spleen. We evaluated whether transient RES blockade through systemic pre-administration of dextran sulfate or liposomes could extend the circulation time and enhance tumor accumulation of siRNA-LNPs in tumor-bearing mice. In two different mouse prostate cancer (PCa) xenograft models, we observed that, upon systemic administration, LNPs still predominantly accumulated in the liver and spleen, with only limited tumor uptake. Our findings demonstrate that pre-treatment with dextran sulfate or liposomes did not enhance siRNA-LNP blood circulation time or tumor accumulation *in vivo*, indicating the need for alternative strategies to enhance siRNA-LNP delivery to tumors.

## Highlights

• Pre-treatment with liposomes or dextran sulfate does not increase blood circulation time of siAR-LNPs in mice.
• Pre-treatment with liposomes or dextran sulfate does not enhance tumor accumulation of siAR-LNPs in mice.
• This is independent of the LNP PEG-lipid type (DMG or DSG).

## Graphical Abstract

**Figure:**
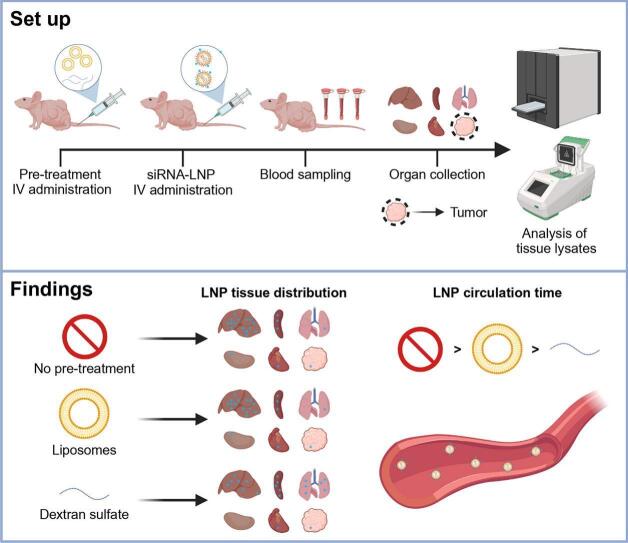

## 1 Introduction

Nanotechnology can enhance the delivery and efficacy of a wide spectrum of anti-cancer agents (van der Meel et al., 2019; Lammers et al., 2016; Bhatia et al., 2022; Gavas et al., 2021). Lipid nanoparticles (LNPs) in particular offer a wide array of possibilities due to their modularity, biocompatibility and capacity to encapsulate various therapeutic payloads, ranging from small molecules to nucleic acids such as small interfering RNAs (siRNAs) (Evers et al., 2018; Hou et al., 2021; Samaridou et al., 2020; Kulkarni et al., 2017; Cullis and Hope, 2017; Pozzi and Caracciolo, 2023). siRNAs hold great potential for targeted therapeutic interventions in oncology by selectively silencing genes of interest (Charbe et al., 2020).

Nevertheless, the effective translation of siRNA-based therapeutics to clinical applications faces significant challenges, with intracellular delivery and concomitant endosomal escape being paramount hurdles (Hu et al., 2020). Encapsulation of siRNAs into LNPs offers a viable solution, protecting the payload from degradation and facilitating cellular entry (Jayaraman et al., 2012). Despite their potential, the clinical translation of nanoparticles for cancer treatment has been hampered by challenges related to their rapid clearance from the bloodstream by the reticuloendothelial system (RES), leading to limited systemic circulation and reduced accumulation at the tumor site (Wilhelm et al., 2016; Zhang et al., 2016; El Moukhtari et al., 2023; Gomes-da-Silva et al., 2012).

The RES plays an essential role in immune surveillance and the clearance of foreign particles from the blood (Iio and Wagner, 1963; Wagner and Iio, 1964), with key cells including Kupffer cells, liver sinusoidal endothelial cells (LSECs), and splenic macrophages (Shi et al., 2011). Upon intravenous administration, most LNPs accumulate in the liver and spleen, where they are taken up by reticuloendothelial system (RES) cells or hepatocytes, restricting their distribution to other tissues (Shi et al., 2011; Sago et al., 2019). As a result, their therapeutic effectiveness at tumor sites may be compromised (El Moukhtari et al., 2023; Gomes-da-Silva et al., 2012).

In order to overcome such limitations, focus has been drawn to the development of new strategies to evade the RES clearance of nanoparticles (Ouyang et al., 2020). By transiently blocking the RES, the blood circulation time of nanoparticles can be extended, thereby increasing the likelihood of tumor accumulation through the enhanced permeability and retention (EPR) effect (Sun et al., 2017). Some of the strategies used to effectively reduce RES clearance of nanoparticles and to increase tumor uptake include: pre-treatment with liposomes (Sun et al., 2017; Proffitt et al., 1983), dextran sulfate (Watson et al., 2016; Patel et al., 1983), chloroquine (Wolfram et al., 2017) or gadolinium (Diagaradjane et al., 2010) to block RES macrophage uptake; depletion of Kupffer cells (Tavares et al., 2017; Ohara et al., 2012); tailoring of nanoparticle size (Charrois and Allen, 2003; Nagayasu et al., 1999) or charge (Juliano and Stamp, 1975); and coating with polyethylene glycol (PEG) (Martin et al., 1994; Li and Huang, 2008) or CD47 (Tang et al., 2019; Hayat et al., 2020) to avoid or delay recognition by the immune system. Moreover, it has been shown recently that exceeding an intravenous bolus dose threshold of 1 trillion nanoparticles in mice improved their delivery to tumors, by saturating Kupffer cell uptake, thereby extending nanoparticle circulation, and ultimately enhancing tumor delivery (Ouyang et al., 2020).

This work aims at transiently blocking the RES to enhance the accumulation of siRNA-LNPs in prostate cancer engrafted tumors in mice. We encapsulated siRNA against the androgen receptor (AR), a key driver in prostate cancer, with the ultimate goal to include AR knockdown as a measure for functional transfection and tumor treatment. Given that empty LNPs are highly inflammatory due to their ionizable lipid component (Ndeupen et al., 2021), priming the animals with nanoparticles containing ionizable lipids was not desirable. We therefore studied two well-established RES blockade strategies—non-PEGylated liposomes and dextran sulfate—both recognized for their potential to reduce the uptake of nanoparticles by the RES (Sun et al., 2017; Proffitt et al., 1983; Watson et al., 2016; Patel et al., 1983; Saunders et al., 2020). Non-PEGylated liposomes may saturate scavenger receptors or endocytic pathways, reducing the internalization by Kupffer cells and LSECs of subsequently administered therapeutic agents (Saunders et al., 2020). Dextran sulfate blocks Scavenger Receptor Class A (SR-A), a key receptor involved in monocyte/macrophage uptake of nanoparticles, and its blockade reduces the clearance of lipid-based nanoparticles, thereby enhancing their circulation time (Watson et al., 2016).

Two LNP formulations were tested in mouse xenograft models engrafted subcutaneously with human prostate cancer (PCa) cell lines. Each of these two formulations contained a different PEG-lipid, PEGylated myristoyl diglyceride (DMG) and PEGylated distearoyl-sn-glycerol (DSG). These two PEG-lipids are known to influence the blood circulation half-lives of LNPs to different extents (Mui et al., 2013a). PEG-DSG contains a phospholipid tail of 18 carbon residues. This stably anchors the PEG onto the LNP surface, which is known to make the particles circulate longer at the cost of making them less effective for RNA transfection. PEG-DMG contains a phospholipid tail of 14 carbon residues, leading to faster shedding and therefore shorter circulation times, but making them more effective for RNA transfection (Mui et al., 2013a; Lee et al., 2016). These LNP formulations were validated close variants of an FDA-approved lipid nanoparticle formulation (Onpattro), which has been the gold standard for siRNA-mediated gene silencing in the liver.

We assessed the circulation times and biodistribution profiles of the administered siAR-LNPs in the tumor and organs. To the best of our knowledge, there are no published studies regarding the impact of liposome and dextran sulfate-mediated RES blockade on tumor accumulation of siRNA-LNPs.

## 2 Materials and methods

### 2.1 Materials

D-Lin-MC3-DMA (VKB, India), cholesterol (Merck, Darmstadt, Germany), 1,2-distearoyl-sn-glycero-3-phosphocholine (DSPC) (Lipoid, Ludwigshafen am Rhein, Germany), 1,2-dioleoyl-sn-glycero-3-phosphoethanolamine (DOPE), 1,2-dimyristoyl-rac-glycero-3-methoxypolyethylene glycol-2000 (DMG-PEG), 1,2-Distearoyl-rac-glycero-3-methylpolyoxyethylene glycol-2000 (DSG-PEG), 1,2-distearoyl-sn-glycero-3-phosphoethanolamine-N-Cyanine 5.5 (18:0 Cy5.5 PE), 1,2-distearoyl-sn-glycero-3-phosphoethanolamine-N-Cyanine 7 (18:0 Cy7 PE) (Avanti Polar Lipids, Alabama, USA), cKK-E12 (Cayman Chemical Company, Michigan, USA) were dissolved in 100 % ethanol (Merck). siRNA androgen receptor, siRNA non-targeting, siRNA androgen receptor-Cy5.5 labeled, were ordered at Integrated DNA Technologies (Iowa, USA) as single strands and then annealed for 5 min at 95 °C. Dextran sulfate sodium salt with a relative molar mass (M_r_) of ∼500,000 was ordered at Merck.

### 2.2 LNP production

Lipid nanoparticles (LNPs) were generated through microfluidic mixing employing the NanoAssemblr Benchtop system (Precision Nanosystems, Vancouver, Canada). LNP synthesis involved the blending of an ethanolic phase containing lipids with an acidic aqueous phase consisting of 100 mM sodium acetate (pH 4.0) containing siRNA. The formulation occurred at a flow rate ratio (aqueous:organic) of 3:1, with a total flow rate of 4.0 ml/min. The lipids were dissolved in 100 % ethanol at a total lipid concentration of 5–20 mM. The LNPs were comprised of D-Lin-MC3-DMA / Cholesterol / DSPC / DMG-PEG or PEG-DSG, with molar percentages of 50 / 37.5 / 10 / 2.5, respectively. The LNPs containing fluorescent lipids contained a molar percentage of 0.2 % in detriment of cholesterol. For the liposome pre-treatment *in vitro* LNP uptake experiment, the following formulation was additionally used: cKK-E12 / Cholesterol / DOPE / DMG-PEG / Cy5–18:0 PE, with molar percentages of 25 / 34.8 / 37.5 / 2.5 / 0.2, respectively. Encapsulation of siRNA was achieved at an N/P ratio (ionizable lipid/RNA) of 6:1. Immediately post-production, LNPs underwent dialysis against an excess of phosphate-buffered saline (pH 7.4) using Slide-a-Lyzer™ dialysis cassettes G2 (membrane cutoff 20 kDa), for a duration of 16–28 h. Following dialysis, LNPs were subjected to sterilization using 0.45 μm PVDF membrane filters and concentrated using Amicon® Ultra-15 centrifugation filter columns with a membrane cut-off of 10 kDa at 2500 x g and 4 °C. The purified LNPs were stored at 4 °C and used within 60 days after production.

### 2.3 Dynamic light scattering for size and polydispersity index

The hydrodynamic diameter of LNPs was assessed using Dynamic Light Scattering (DLS) with a Zetasizer Nano S instrument (Malvern Panalytical, Malvern, UK) featuring a 4 mW HeNe laser emitting at 633 nm. LNPs were diluted in Dulbecco's Phosphate-Buffered Saline (DPBS, pH 7.4), and scattering measurements were conducted at 25 °C at a 173° angle and for a duration of 10 s, repeated 10 times. These series of measurements were repeated three times for each specimen.

### 2.4 Zeta potential

Zeta potential was determined using the Zetasizer Nano Z (Malvern Panalytical). Before measuring the samples, the device was calibrated using a Zeta Potential Transfer Standard (Malvern Panalytical). LNPs were diluted in 10 mM HEPES (pH 7.4) and each sample underwent at least three independent measurements.

### 2.5 Nanoparticle tracking analysis (NTA)

Nanoparticle Tracking Analysis (NTA) was conducted on a NanoSight NS500 (Malvern Panalytical). The samples were appropriately diluted in DPBS, pH 7.4 to achieve a suitable particle concentration. After loading into the sample chamber, the camera level was set at 16, and the sample was measured three times for a duration of 30 s each. Subsequent analysis was performed using Nanosight NTA 3.4 software with a detection threshold of 7.

### 2.6 siRNA quantification and encapsulation efficiency assessment

Total RNA concentration was quantified using the Quant-It™ Ribogreen RNA Assay kit (Thermo Fisher Scientific, Waltham, MA, USA) in the presence of 2 % (*v*/v) Triton® X-100 (Merck) in TE buffer. The concentration of free RNA was determined in TE buffer. The fluorescent signal from lysed LNPs represented the total RNA (μg/μl) while that from non-lysed samples indicated free RNA (μg/μl). To quantify RNA concentration, reference calibration curves were established using both 2 % Triton® X-100 and TE buffer. The encapsulated RNA concentration was determined by subtracting the free RNA concentration from the total RNA concentration. The percentage of encapsulated RNA was then determined using the formula: ((Total RNA – Free RNA) / Total RNA) x 100.

### 2.7 Cell culture conditions

PC3, DU145 and Hepa 1–6 cells were cultured in Dulbecco's Modified Eagle Medium (DMEM) supplemented with l-Glutamine (Thermo Fisher Scientific) and 10 % fetal bovine serum (FBS) (Merck). LNCaP and J774A.1 cells were cultured in Roswell Park Memorial Institute (RPMI) 1640 Medium (Thermo Fisher Scientific), also supplemented with 10 % FBS. PC346C cells were cultured in Dulbecco's modified Eagle's medium/Ham's F12 (DMEM/F12 1:1) containing the following supplements: BSA (fraction V) 0.01 % (Merck), FBS 2 % (Merck), EGF 10 ng/ml (Merck), ITS-G 1 % (Thermo Fisher Scientific), Hydrocortisone 0.5 μg/ml (Merck), Triiodothyronine (T3) 1 nM (Merck), Phosphoethanolamine 0.1 mM (Merck), Cholera toxin 50 ng/ml (Merck), Fibronectin 0.1 μg/ml (Merck), Fetuin 20 μg/ml (Merck), R1881 0.1 nM (Merck). All cell lines were cultured in the presence of 100 μg/ml streptomycin and 100 U/ml penicillin (Thermo Fisher Scientific) under standard conditions at 37 °C and 5 % CO_2_. LNCaP, PC3 and DU145 were cultured in a Biosafety level 1 (BSL1) laboratory whereas PC346C was cultured in a Biosafety level 2 (BSL2) laboratory in Corning® Primaria™ Cell Culture Flasks (Corning Inc., Massachusetts, USA). The PC346C cell line was kindly provided by Wytske van Weerden from Erasmus Medical Center Rotterdam, the Netherlands.

### 2.8 Androgen receptor (AR) *in vitro* gene silencing

A total of 100,000 cells per well were seeded in a 12-well plate, 24 h prior to transfection. LNPs containing 0.5 μg/ml encapsulated siRNA were added to PC346C, LNCaP, DU145 and PC3. RNA and protein were extracted 24 h and 48 h after LNP addition, respectively. For RNA extraction, the medium was discarded, the cells were washed with cold PBS, and 1000 μl of TRIzol (Thermo Fisher Scientific) were added to each well. Next, the lysates were resuspended and stored at −80 °C. For protein extraction, the medium was discarded and the cells were washed with cold PBS, then 350 μl RIPA buffer 1× (Thermo Fisher Scientific) supplemented with Protease Inhibitor Cocktail (Merck) was added to the wells. Next, the wells were scraped with a cell scraper and the lysate was resuspended and kept on ice for 30 min. After that the lysates were centrifuged at 16,000 x*g* for 20 min at 4 °C, the supernatants were collected and stored at −80 °C.

### 2.9 mRNA and small RNA isolation from cells and tissues

Following sample thawing at room temperature and transfer to 1000 μl TRIzol, 200 μl of chloroform was added to each tube containing TRIzol, and vigorous shaking ensued for 15 s. After a 2-min incubation period at room temperature, centrifugation was performed at 4 °C, 12,000 x g for 15 min. The resulting aqueous phase was then carefully extracted and transferred to RNase free Eppendorf tubes containing 1 μl of Glycoblue™ coprecipitant (Thermo Fisher Scientific). Subsequently, 500 μl of cold isopropanol was added to the tubes, thoroughly mixed and incubated for 10 min. Further centrifugation at 4 °C, 12,000 x g for 15 min (mRNA) or 30 min (siRNA) followed, and the resulting isopropanol/chloroform mixture was discarded. After a quick spin and removal of remaining liquid with finer tips, 1 ml of 75 % (mRNA) or 80 % (small RNA) ethanol (prepared by diluting absolute ethanol with nuclease free water) was added, followed by vortexing for 10 s. The samples were then centrifuged at 7500 x*g* for 5 min at 4 °C, and the ethanol was removed. After a brief spin, residual ethanol was eliminated, and the pellets were air-dried for 10 min. The samples were placed on ice at this stage, and the pellets were reconstituted in 40 μl nuclease-free water, before freezing at −80 °C.

### 2.10 RT-qPCR analysis of AR mRNA expression

The isolated RNA was quantified by DS-11 Spectrophotometer (DeNovix, Delaware, USA) and it was converted to cDNA using the iScript™ cDNA Synthesis Kit (Bio-Rad) and following manufacturer's instructions. The PCR vials containing 750 ng of RNA, nuclease free water, iScript Reaction mix (iScript mix) and iScript Reverse Transcriptase (iScript RT) in a total volume of 20 μl per sample were kept on ice. The PCR vials were then placed in a C1000 Touch Thermal Cycler (Bio-Rad) for 5 min at 25 °C, 20 min at 46 °C, 1 min at 95 °C, and at 4 °C until further processing. They were then diluted 1:5 in nuclease-free water and underwent qPCR analysis with iQ SYBR Green Supermix (Bio-Rad) in a CFX96 Real-Time PCR Detection System (Bio-Rad). The conditions for qPCR were as follows: 3 min at 95 °C and 40 cycles (15 s at 95 °C and 40 s at 60 °C) with a melting curve 55–95 °C in 0.5 °C increments. The changes in gene expression of AR mRNA were quantified relative to the expression of GAPDH mRNA and each sample was run in triplicate. The primer sequences used were the following: GAPDH: Forward: 5’-ACAGTCAGCCGCATCTTC-3′, Reverse: 5’-GCCCAATACGACCAAATCC-3′. Androgen Receptor: Forward: 5’-GTAACTACCCGAGCATGGC-3′, Reverse: 5’-CCCCTTGTAGTGGGTCAAAC-3′.

### 2.11 Western blot analysis of AR protein expression

Protein concentrations were determined from the RIPA lysates by employing a Pierce™ BCA protein assay (Thermo Fisher Scientific), following the manufacturer's guidelines. Samples were mixed with sample loading buffer containing 100 μM DTT, followed by heat-inactivation at 95 °C for 5 min. Next, the prepared samples were loaded into 4–12 % gradient Bis-Tris polyacrylamide gels (Thermo Fisher Scientific) and were separated by gel electrophoresis. After electrophoresis, proteins were transferred onto 0.45 μm-pore Immobilon®-FL PVDF activated membranes (Merck). The membranes were then incubated overnight at 4 °C in Odyssey Blocking Buffer (LI-COR Biosciences) mixed (1:1) with Tris buffered saline (TBS). The membranes were stained using the following antibodies: rabbit recombinant Anti-Androgen Receptor antibody [ER179(2)] (Abcam, ab108341, 1:5000) and mouse anti-β-actin (Cell Signaling, 8H10D10, 1:1000). As secondary antibodies, anti-rabbit IgG-AlexaFluor 680 (Thermo Fisher Scientific, A-21076) and IRDye® 800CW Donkey Anti-Mouse IgG (LI-COR Biosciences, 926–32,212) were used at a 1:5000 dilution. All antibody dilutions were carried out in Odyssey Blocking Buffer (LI-COR Biosciences) mixed (1:1) with TBS-Tween 0.1 %. Protein visualization was performed using an Odyssey Infrared Imager (LI-COR Biosciences) with detection at 700 and 800 nm. AR protein knockdown was evaluated normalized to β-actin expression.

### 2.12 Ethical statement on animal experiments

All animal experiments were conducted in accordance with the ethical standards and regulations set forth by the Utrecht Animal Welfare Body and complied with the Dutch Experiments on Animals Act (WOD) licenses AVD108002016544 and AVD10800202115026. The experiments adhered to the guidelines outlined in the “Guide for the Care and Use of Laboratory Animals”. The animals had continuous access to water and standard chow *ad libitum*, and were kept in an environment with a 12-h light/dark cycle. For the experiments, the mice were randomly assigned to different treatment groups and cages and blinding of the operators was carried out when possible.

### 2.13 Biodistribution in PC346C engrafted animals

After a 7-day acclimatization period, male outbred nude mice-NU/NU (Crl:NU-Foxn1nu) (Charles River Laboratories, 25–33 g, 7–10 weeks) were subcutaneously injected in the right flank with 5 × 10^6^ PC346C tumor cells in 100 μl PBS. Tumor growth was monitored until reaching 150 mm^3^, when treatments were administered intravenously (i.v.) *via* the tail vein. The first injection contained liposomes (360 mg/kg), dextran sulfate (Merck) (30 mg/kg) or PBS (control). Ten minutes after liposomes administration and 2 h after dextran sulfate or PBS administration, a second i.v. injection consisted of PEG-DSG LNPs containing AR-encoding siRNA, at a dose of 5 mg/kg siRNA. LNPs were labeled with 0.2 % of total lipid content being 18:0 Cy5.5 PE (Avanti Polar lipids). Collection of approximately 70 μl of blood samples from left and right submandibular punctures was scheduled at 1 min and 1 h after LNP injection. Four hours after LNP injection, the animals were euthanized *via* intraperitoneal (i.p.) overdose of 2.1 mg pentobarbital in 0.9 % NaCl, blood was collected after eye removal, the tumor and organs were perfused by cardiac perfusion with PBS and finally excised for analysis. The tumor, liver, spleen, heart, lungs, kidneys, and brain of the animals were collected and placed in containers on ice for imaging analysis. Full organ imaging analysis was conducted using a Pearl Impulse Imager (LI-COR Biosciences, Nebraska, USA). Immediately after imaging, organs were snap-frozen in liquid nitrogen and stored at −80 °C. The blood samples, collected in serum separation tubes (Sarstedt AG & Co. KG, Nümbrecht, Germany), were centrifuged at 10,000 x*g* for 5 min at 4 °C and the serum was stored at −80 °C until further analysis.

### 2.14 Biodistribution in LNCaP engrafted animals

After a 7-day acclimatization period, male NMRI-nu Immunodeficient mice (Rj:NMRI-Foxn1nu/nu), (Janvier Laboratories, 32–40 g, 7–10 weeks) were subcutaneously injected in the right flank with 2.5 × 10^6^ LNCaP tumor cells in 200 μl (50 %RPMI +50 % Corning™ Matrigel™ GFR Membrane Matrix (Thermo Fisher Scientific)). Tumor growth was monitored until it reached 150 mm^3^, when treatments were administered i.v. in the tail vein. The first injection contained liposomes (360 mg/kg) or PBS (control). After 10 min, a second injection consisted of PEG-DMG or PEG-DSG fluorescently labeled LNPs at a dose of 2.5 mg/kg siRNA. LNPs were dually labeled with 0.2 % of total lipid content being 18:0 Cy7 PE (Avanti Polar lipids) and 10 % of AR siRNA labeled with Cy5.5 (Integrated DNA technologies). Groups treated with PEG-DMG LNPs, had around 40 μl of blood collected 1 min, 1 h, and 4 h after the LNP i.v. injection *via* vena saphena using K2/EDTA glass capillaries (purple) (Vitrex Medical A/S, Herlev, Denmark). Similarly, groups treated with PEG-DSG LNPs, had around 40 μl of blood collected 1 min, 1 h, 4 h, and 6 h after the LNP injection *via* vena saphena using K2/EDTA glass capillaries. Six hours (PEG-DMG LNPs) or 24 h (PEG-DSG LNPs) post-LNP injection, the animals were anesthetized *via* i.p. administration of 2.1 mg pentobarbital in 0.9 % NaCl, blood was collected after eye removal using K2/EDTA glass capillaries, the organs were perfused by cardiac perfusion with PBS and collected for analysis. Tumor, liver, spleen, heart, lungs, and kidneys, were collected and placed in containers on ice for full organ imaging analysis of both fluorophores. Full organ imaging analysis was conducted using a Pearl Impulse Imager (LI-COR Biosciences). Immediately after imaging, organs were snap-frozen in liquid nitrogen and stored at −80 °C. The plasma was collected after centrifugation at 1200 x*g* for 15 min at 4 °C and stored at −80 °C until further analysis.

### 2.15 Tissue lysates preparation for fluorescence and protein analysis

Serum and plasma samples were initially diluted 2-fold in PBS, followed by the transfer of 25 μl into a black 384-well plate and fluorescence was measured. Tissue lysates were prepared from tumor, liver, spleen, lungs, kidneys, heart and brain. Organs were thawed and weighed and approximately 100 mg of each organ was transferred to a screw cap 2 ml micro tube (Sarstedt) containing ceramic beads (1.4 mm) (Qiagen, Hilden, Germany). Next, 5 μl of 1× RIPA buffer with protease inhibitors were added for every milligram of tissue. Homogenization was carried out using a Mini bead-beater (Bertin Technologies, Montigny-le-Bretonneux, France) for 60 s at 5000 rpm. After centrifugation for 10 min at 10,000 x g and 4 °C, the supernatant was collected and 25 μl transferred to a black 384-well plate, and fluorescence was measured. Regarding tissue lysates, results were expressed as fluorescence per milligram of tissue normalized to the administered dose. The concentration of LNPs in blood serum/plasma was presented as a percentage of the fluorescence measured right after injection. Fluorescence from both plasma/serum and tissue lysates was analyzed using a Spectramax ID3 instrument (Molecular Devices, California, USA) at 675/720 nm (Cy5.5) and 750/790 nm (Cy7) for excitation/emission wavelengths, respectively. Androgen receptor protein analysis was carried out by Western Blot as previously described.

### 2.16 Tissue lysates preparation for RNA analysis

Tissue lysates were prepared from tumor, liver, spleen, lungs, kidneys, heart and brain. Organs were thawed and weighed and approximately 100 mg of each organ was transferred to a screw cap 2 ml micro tube (Sarstedt) containing ceramic beads (1.4 mm) (Qiagen). For RNA extraction, 10 μl of TRIzol was added for every milligram of tissue tube and homogenization was carried out using a Mini bead-beater (Bertin Technologies) for 60 s at 5000 rpm. After centrifugation for 10 min at 10,000 x g and 4 °C, the supernatant was collected. mRNA/siRNA isolation, quantification and RT-qPCR analysis was carried out as described in the previous sections. For AR siRNA detection, blank liver tissue lysates were spiked with a serial dilution of siRNA concentrations to create a calibration curve in order to accurately correlate the changes in concentration with the qPCR Ct values.

### 2.17 RT-qPCR analysis for the detection of AR siRNA in tissue lysates

The isolated RNA was quantified by DS-11 Spectrophotometer (DeNovix) and it was converted to cDNA using the Taqman™ MicroRNA Reverse Transcription Kit (Thermo Fisher Scientific) and following manufacturer's instructions. The PCR vials were placed on ice and contained 4 μl of the Taqman™ mix and 3.5 μl of RNA template. A no-RNA template control reaction was included as a negative control for the siRNA detection. The PCR vials were then placed in a C1000 Touch Thermal Cycler (Bio-Rad) for 30 min at 16 °C, 30 min at 42 °C, 5 min at 85 °C and at 4 °C. The samples were then diluted 1:25 in nuclease-free water and underwent qPCR analysis with iQ SYBR Green Supermix (Bio-Rad) in a CFX96 Real-Time PCR Detection System (Bio-Rad). The conditions for qPCR were as follows: 3 min at 95 °C and 50 cycles (15 s at 95 °C + 30 s at 60 °C + 30 s at 72 °C) with a melting curve 55–95 °C in 0.5 °C increments. Each sample was run in triplicate. The stem-loop primer sequence designed to amplify siRNA AR for the Taqman™ MicroRNA Reverse Transcription was the following: 5’-GTCGTATCCAGTGCAGGGTCCGAGGTATTCGCACTGGATACGACAACTGG-3′. The primers used for the subsequent qPCR reaction were as follows: Forward-siAR: 5’-CCGCTATGGGCTTGACTTTC-3′, Reverse-Universal: 5’-CCGCTATGGGCTTGACTTTC-3′.

### 2.18 *In vitro* experiments with liposomes

In separate 48-well plates, 30,000 J774A.1 and Hepa 1–6 cells were seeded. After 48 h, liposomes were added to the wells at a lipid concentration of 0.5 mg/ml for 10 min or 4 h in full medium. Following removal of the medium, Cy5-LNPs were added at a concentration of 100 ng encapsulated RNA per well. The cells were then incubated for 2 h at 37 °C and 5 % CO_2_. J774A.1 cells were washed with DPBS twice, and after addition of 250 μl DPBS 2 % FBS the wells were scrapped with the back of a pipette tip. As for Hepa 1–6 cells, the cells were washed twice with DPBS, trypsinized and resuspended in 250 μl DPBS 2 %FBS. The well content from both J774A.1 and Hepa 1–6 cells was transferred to 96 U-bottom well plates (Greiner) where uptake was analyzed on a FACS Canto II flow cytometer (Becton, Dickinson and Company, New Jersey, US).

### 2.19 Statistical analysis

Statistical analyses were conducted using GraphPad Prism v10 (GraphPad Software, California, USA).

## 3 Results

### 3.1 siRNA-LNPs formulation and validation of *in vitro* knockdown of the androgen receptor

**Fig. 1:**
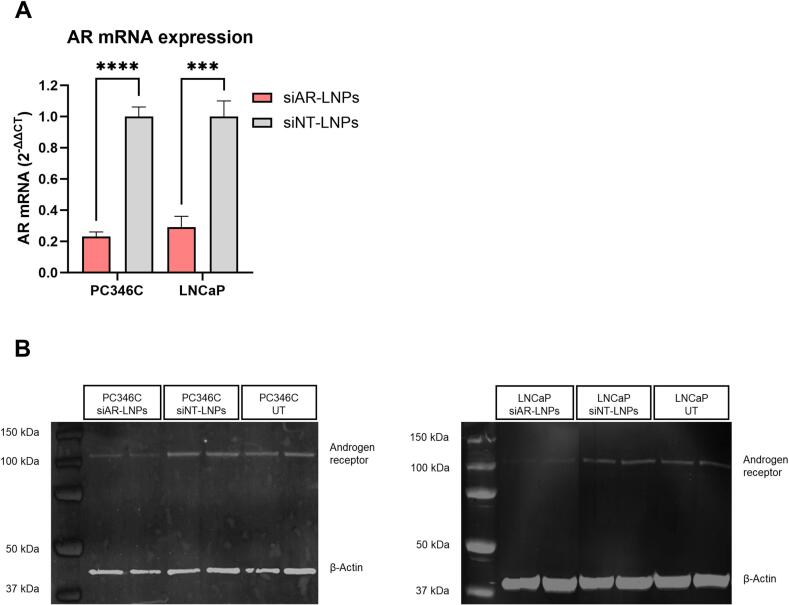
siAR-LNPs knock down AR *in vitro* in PC346C and LNCaP as demonstrated by RTqPCR and Western Blot (WB). PEG-DMG siRNA-LNPs (0.5 μg/ml per well) added to 12-well plates. (A) Expression normalized to GADPH for RTqPCR 24 h after LNP addition (B) and normalized to β-actin for WB 48 h after LNP addition. For RTqPCR data represent mean ± SD (*n* = 3 biological replicates), and significance of the differences between groups was determined by a two-tailed unpaired *t*-test. ****, *p*-value <0.0001; ***, *p*-value <0.001. For WB: *n* = 2 biological replicates.

We started by defining our LNP formulation details based on previously published studies where siAR-LNPs were systematically administered to mice engrafted with prostate cancer tumors (Quick et al., 2022; van der Meel et al., 2021). The siRNA-LNPs we prepared were composed of D-Lin-MC3-DMA / Cholesterol / DSPC / PEG-DMG, with molar percentages of 50 / 37.5 / 10 / 2.5, respectively. After successful characterization of siRNA-LNPs by size (∼70 nm diameter), polydispersity index (< 0.3), zeta potential (∼neutral), and siRNA encapsulation efficiency (> 99 %) (Table S1), we decided to validate, as a proof of concept, whether our siAR-LNPs could knock down the AR gene *in vitro*, both at the mRNA (Fig. 1**a**) and protein levels (Fig. 1**b**), in two prostate cancer cell lines that express the AR wild type (PC346C and LNCaP). Both the levels of AR mRNA (Fig. 1a) and AR protein (Fig. 1b) were significantly reduced in PC346C (77 % mRNA knockdown) and LNCaP (71 % mRNA knockdown) upon treatment with PEG-DMG siAR-LNPs, compared to PEG-DMG non-targeting siRNA LNPs (siNT-LNPs). This demonstrated the efficacy of the siRNA. The PEG-DMG formulation has been developed for liver applications, whereas the inclusion of PEG with longer lipid tails has been shown to decelerate liver uptake (Mui et al., 2013b), which could favor extrahepatic applications. Therefore, we also assessed AR mRNA levels in LNCaP cells 24 h after treatment with the same formulation, but replacing PEG-DMG for PEG-DSG. We observed a 31 % mRNA knockdown (Fig. S1). Although this formulation showed less AR gene silencing *in vitro*, it has been previously reported that LNP-mediated RNA delivery *in vitro* poorly predicts *in vivo* delivery (Paunovska et al., 2018). We decided to study the biodistribution of this PEG-DSG LNP formulation *in vivo* using a murine prostate cancer xenograft model because it had demonstrated improved LNP tumor accumulation in a previously published prostate cancer study (Quick et al., 2022).

### 3.2 Pre-treatment with liposomes or dextran sulfate does not enhance siRNA-LNP circulation time *in vivo*

**Fig. 2:**
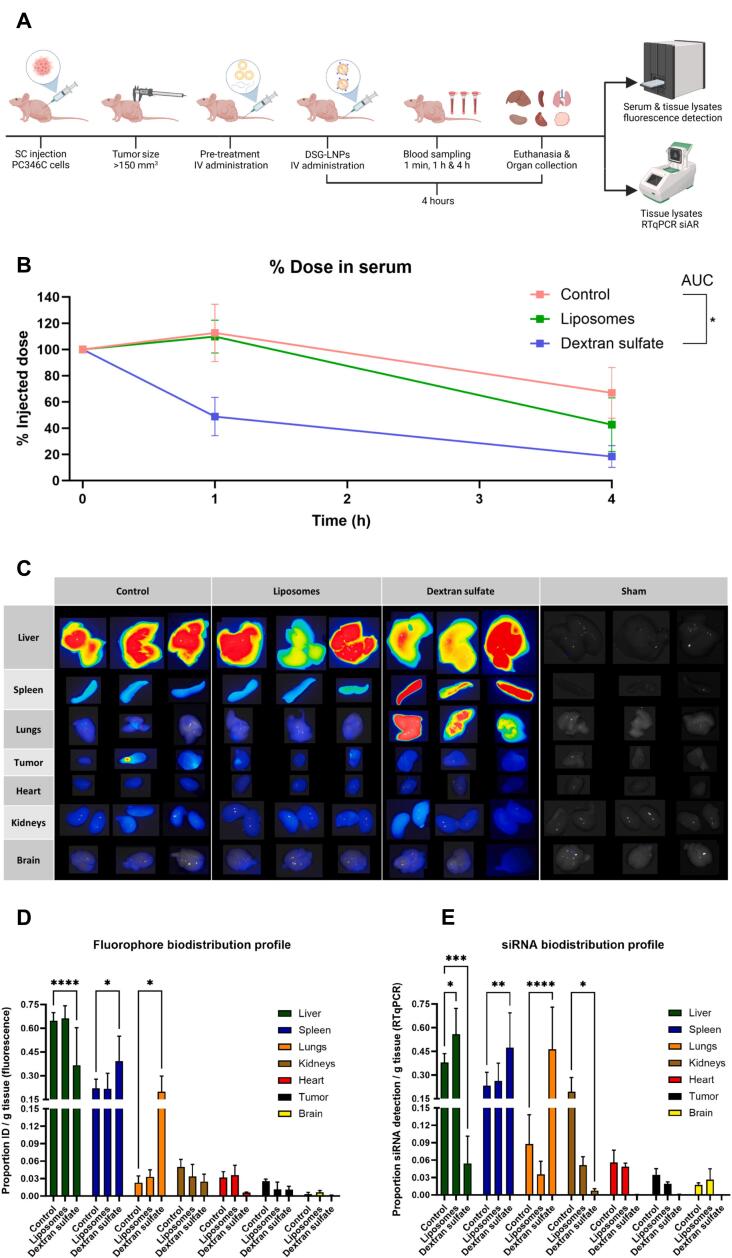
Liposome and dextran sulfate pre-treatments do not enhance circulation time or tumor accumulation of systemically administered siAR-LNPs in mice. (A) Overview of the general experimental workflow for RES blockade *in vivo* experiments. NU/NU mice bearing PC346C tumors were systemically injected with dextran sulfate (30 mg/kg), liposomes (360 mg/kg) or PBS (control), followed by i.v. injection of siAR-PEG-DSG-LNPs containing 0.2 % DSPE-Cy5.5 at a dose of 5 mg/kg siRNA. Blood samples were collected *via* submandibular puncture (1 min and 1 h) and cardiac puncture (4 h), and Cy5.5 signal in serum was quantified by fluorescent spectroscopy. After 4 h, mice were sacrificed, organs perfused with PBS and tissues were collected for Cy5.5 and siAR quantification. (B) siAR-LNPs detection in serum at multiple time points as determined by Cy5.5 signal. The serum concentration is represented as a percentage of the directly measured serum fluorescence in individual animals following injection (*t* = 1 min). (C) Whole-organ fluorescence spectroscopy measured by Cy5.5 signal 4 h after siAR-LNP administration. Measurement of Cy5.5 signal (D) and RTqPCR quantification of siAR (E) in tissue lysates. Biodistribution of siAR-LNPs, both by Cy5.5 and siAR quantification is expressed as percentage of the injected dose per gram (% ID/g) of tissue. For the statistical analysis of siAR-LNPs detection in serum, group comparisons were conducted through a One-Way ANOVA Dunnett's T3 multiple comparisons test by comparing the areas under the curve (AUC) of the treatment groups. For the biodistribution studies, both by fluorophore and siAR detection, a Two-Way ANOVA with Dunnet correction for multiple comparisons test was performed comparing the mean signals from each organ between the treatment groups and the control group. Data represent mean ± SD (*n* = 3 animals). ****, *p*-value <0.0001; ***, *p*-value <0.001; **, *p*-value <0.01; *, *p*-value <0.05; blank/ns: no significant difference. SC: subcutaneous, IV: intravenous, siAR: siRNA Androgen Receptor, AUC: Area under the curve.

We conducted a biodistribution study of siAR-LNPs in athymic nude mice subcutaneously engrafted with PC346C tumors. Since this previous study with siAR-LNPs (Quick et al., 2022) demonstrated that most of the dose was still cleared by liver and spleen, we additionally investigated whether pre-treatment with liposomes or dextran sulfate could enhance LNP accumulation in the tumor. For this experiment, we prepared and characterized siAR-LNPs labeled with a Cy5.5 fluorescent lipid (Table S1) and validated that the LNPs and liposomes we used were stable at 4 °C over the course of 60 days (Fig. S2). The experimental plan to quantitatively assess the pharmacokinetic characteristics and biodistribution of siAR-LNPs with and without pre-treatment of liposomes or dextran sulfate is shown in Fig. 2a. As we aimed to combine the RES blockade strategy with the nanoparticle dose threshold for tumor delivery (Ouyang et al., 2020), we determined by nanoparticle tracking analysis (NTA) that the liposome treated animals were intravenously injected with ∼1.2 × 10^14^ liposomal particles, approximately two orders of magnitude above the suggested 10^12^ particles threshold for enhanced tumor accumulation (Ouyang et al., 2020).

We first determined the effect of liposomes and dextran sulfate pre-treatment on the circulation time of PEG-DSG LNPs by measuring the Cy5.5 fluorescent signal in serum at multiple time points after injection. The percentage of the LNP dose (% ID) remaining in circulation after 4 h was similar for the liposome group compared to the control, but significantly lower for the dextran sulfate group (Fig. 2b). When comparing the areas under the curve (AUC) of LNP-serum detection for the different treatments over the course of 4 h, the calculated AUCs were 375.7 (A.U.) for the control group, 333.7 (A.U) for the liposome and 175.3 (A.U.) for the dextran sulfate group. Hence, liposome pre-treatment did not enhance LNP circulation time and dextran sulfate, surprisingly, reduced the circulation time of the tested LNPs (Fig. 2b). When analyzing these results, we hypothesized that the reduced LNP circulation time in the dextran sulfate group might be caused by LNP aggregation due to electrostatic interactions. To test this, we examined whether LNPs aggregate in the presence of dextran sulfate and demonstrated that LNPs remain stable when mixed with dextran sulfate in PBS at room temperature (Fig. S3).

### 3.3 Priming with liposomes or dextran sulfate does not enhance siRNA-LNP tumor accumulation in PC346C engrafted mice

Next, tissue distribution was assessed 4 h post-administration through whole organ imaging (Fig. 2c), fluorescence spectroscopy of tissue lysates (Fig. 2d), and by RTqPCR detection of siAR in tissue lysates (Fig. 2e). The control and liposome groups showed a very similar biodistribution pattern, where most of the LNP signal is detected in the liver and spleen (Fig. 2c-e). On the other hand, pre-treatment with dextran sulfate significantly shifted LNP accumulation from the liver to the spleen and lungs (Fig. 2c-e). With respect to tumor accumulation, all groups showed equal accumulation in the tumor (Fig. 2c,d). The tumor accumulation percentage of the control group LNPs (∼2.5 % ID/g tissue) is consistent with reported results from a study that investigated similar LNPs in mice with subcutaneously engrafted prostate cancer (van der Meel et al., 2021). We also analyzed the siRNA levels in tissue lysates by RT-qPCR (Fig. 2e). This yielded comparable patterns to those observed for the Cy5.5-labeled lipid of the LNPs and supported the obtained results (Fig. 2d,e).

### 3.4 re-treatment with liposomes reduces the circulation times of PEG-DMG and PEG-DSG siRNA-LNPs *in vivo*

Given that ∼67 % of the LNPs from the control group were still in circulation after 4 h (Fig. 2b), we reasoned that further studies at later time points would be more adequate. Furthermore, we argued that perhaps the effect of RES blockade on long-circulating particles would be less pronounced than for short-circulating particles. For this purpose, we decided to study the biodistribution of PEG-DMG-LNPs (short circulating) at 6 h and PEG-DSG-LNPs (long circulating) at 24 h in follow-up experiments. On top of that, we decided to employ a dual labeling approach by which a lipid in the LNP formulation is labeled with Cy7 and a part of the loaded siAR is labeled with Cy5.5. Moreover, we switched the engrafted prostate cancer model to LNCaP, as s.c. injection of PC346C resulted in only 41 % tumor engraftment. Their slow growth was not ideal for *in vivo* studies, where large cell numbers are required. Finally, we decided to abandon the dextran sulfate studies as dextran sulfate-treated mice showed clear signs of toxicity (shivering and reduced movement in treated mice) and dextran sulfate caused prolonged bleeding of the animals after submandibular puncture due to its anti-coagulant effect (Forwell and Ingram, 2011).

**Fig. 3:**
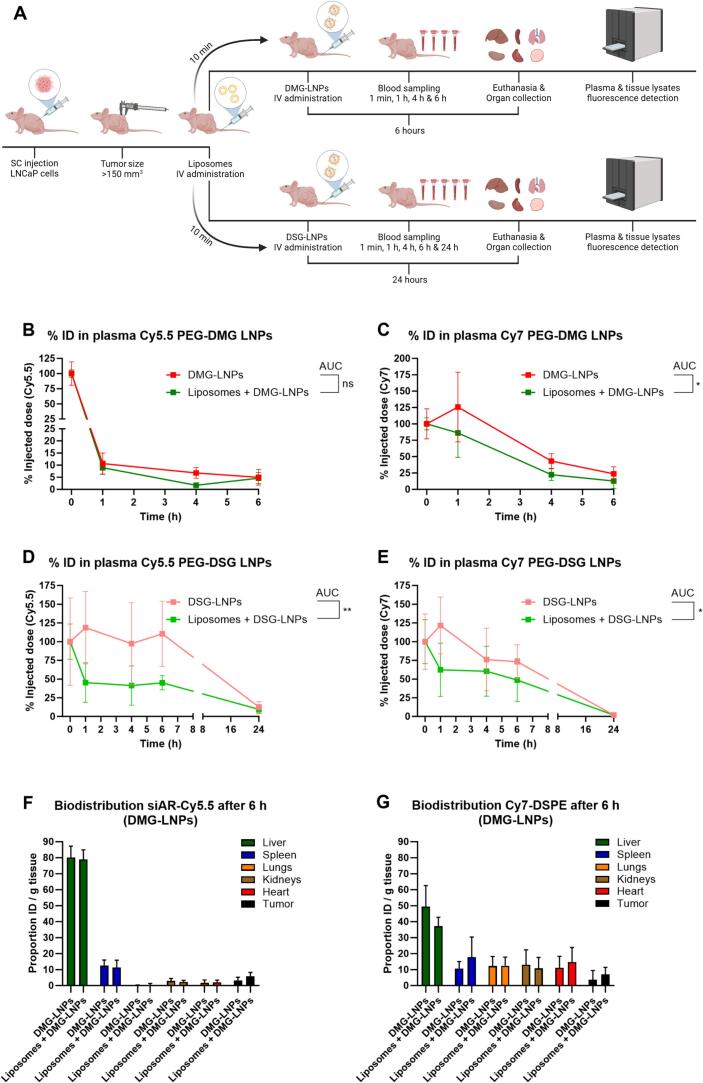

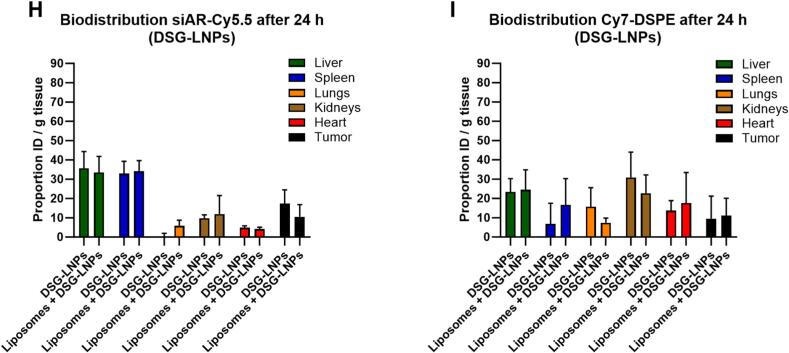
Liposome pre-treatment does not enhance the circulation time or tumor accumulation of systemically administered PEG-DMG and PEG-DSG siRNA-LNPs in mice. (A) NMRI-nu immunodeficient mice bearing LNCaP tumors were systemically injected with liposomes (360 mg/kg) or PBS (control), followed by i.v. injection of dually labeled PEG-DMG or PEG-DSG siAR-LNPs at a dose of 2.5 mg/kg siRNA. Six hours after PEG-DMG-LNPs and 24 h after PEG-DSG-LNPs administration mice were sacrificed, organs perfused with PBS and tissues were collected for Cy5.5 (siRNA) and Cy7 (lipid) quantification. Plasma fluorescence was quantified for siAR-Cy5.5 (B) and Cy7-DSPE (C) at 1 min, 1 h, 4 h and 6 h after PEG-DMG-LNPs treatment. Plasma fluorescence was measured for siAR-Cy5.5 (D) and Cy7-DSPE (E) 1 min, 1 h, 4 h, 6 h and 24 h after PEG-DSG-LNPs treatment. Tissue lysates' fluorescent signals of Cy5.5 (F) and Cy7 (G) were quantified 6 h after PEG-DMG-LNPs treatment. Tissue lysates' fluorescent signals of Cy5.5 (H) and Cy7 (I) were measured 24 h after PEG-DSG-LNPs treatment. The plasma concentration is represented as a percentage of the directly measured plasma fluorescence following injection (*t* = 1 min). siAR-LNPs biodistribution, both by Cy5.5 and by Cy7, is expressed as percentage of the injected dose per gram (% ID/g) of tissue. For the statistical analysis of siAR-LNPs detection in plasma (B-E), group comparisons were conducted by unpaired two-tailed *t*-tests by comparing the areas under the curve (AUC) of the treatment groups. For the biodistribution studies (F-I), a two-way ANOVA with Šidák correction for multiple comparisons test was performed comparing the mean signals from each organ between the treatment groups. Data represent mean ± SD (*n* = 4–5 animals). **, *p*-value <0.01; *, *p*-value <0.05; ns/blank: no significant difference. i.v.: intravenous, AR: androgen receptor, AUC: Area under the curve.

After preparation and characterization of the LNPs (Table S1), we conducted a quantitative evaluation of the pharmacokinetics and biodistribution profile of PEG-DMG and PEG-DSG siAR-LNPs, with and without pre-treatment of liposomes. For animals treated with PEG-DMG LNPs (short-circulating particles) we fixed an endpoint at 6 h and for animals treated with PEG-DSG-LNPs (long-circulating particles) at 24 h post injection (Fig. 3a).

We first determined the circulation time of the LNPs by measuring the Cy5.5 and Cy7 fluorescent signal in plasma at multiple time points. For PEG-DMG-LNPs, the comparison of AUC over the course of 6 h showed a slightly reduced LNP circulation time in the liposome-treated animals compared to the control group for the siRNA-Cy5.5 readout (Fig. 3b) (*p*-value: 0.06). For the labeled lipid (DSPE-Cy7) it was significantly reduced (Fig. 3c). As for the PEG-DSG-LNPs, the %ID in circulation over the course of 24 h was reduced for the liposome treated animals when compared to the control group both for siRNA (Fig. 3d) and LNP (Fig. 3e) fluorescence readouts. Liposomes did not enhance the circulation time of PEG-DMG or PEG-DSG siRNA-LNPs but show reduced circulation times. These results also pointed out the differences between Cy5.5 (siRNA) and Cy7 (lipid) plasma signals over time. It is known that use of lipid fluorescent dyes to track nanoparticles *in vivo* can give unreliable results as they are in many cases unstable and can detach from the nanoparticle and transfer to cells, lipoproteins or other serum components (Münter et al., 2018; Huang et al., 2023). It is for this reasons that the encapsulated active pharmaceutical ingredient (API), in this case the siAR-Cy5.5, acts as our main LNP biodistribution readout. It is noteworthy that the results obtained with tracing of DSPE-Cy7 in PEG-DSG-LNPs for the first 4 h (Fig. 3e), are in line with the circulation results of the previous experiment (Fig. 2b) where DSPE-Cy5.5 was used as a label for PEG-DSG-LNPs.

### 3.5 Priming with liposomes does not enhance PEG-DMG or PEG-DSG siRNA-LNP tumor accumulation in LNCaP engrafted mice

As for the tissue distribution, this was assessed 6 h post-administration for PEG-DMG-LNPs and 24 h post-administration for PEG-DSG-LNPs. Whole-organ fluorescence spectroscopy was measured for Cy5.5 and Cy7, right after organ collection (Fig. S4). Fluorescence spectroscopy of tissue lysates from PEG-DMG-LNP-treated animals revealed no statistically significant differences in tumor accumulation but showed a possible trend towards more tumor accumulation when animals were pre-treated with liposomes for both Cy5.5 (control 3.17 % *vs* liposomes 5.92 %) (Fig. 3f) and Cy7 (control 3.61 % *vs* liposomes 7.04 %) (Fig. 3g). Regarding PEG-DSG-LNPs, there were again no statistically significant differences regarding tumor accumulation for both Cy5.5 (Fig. 3h**)** and Cy7 analysis (Fig. 3i). Following our main biodistribution readout (siAR-Cy5.5) for PEG-DMG and PEG-DSG LNPs, the control and liposome groups showed a very similar biodistribution pattern, where most of the signal was detected in the liver and spleen (Fig. 3f,h). Regarding the Cy7-lipid readouts, most of the signal from PEG-DMG LNP tissue lysates came from the liver and the biodistribution profiles of the control and the liposome groups were very similar (Fig. 3g). In the case of PEG-DSG LNP treated animals, the Cy7 signals from tissue lysates at 24 h showed a broad distribution across the tested organs with particularly high kidney signal, potentially indicating LNP processing by that time and Cy7-lipid label accumulation in the kidney due to its small size (<1.5 kDa) (Fig. 3i).

Priming the animals with liposomes reduced blood circulation of PEG-DMG- and PEG-DSG siRNA-LNPs and did not significantly enhance their tumor accumulation. Moreover, we performed RTqPCR on the AR mRNA in the tumor lysates of the PEG-DSG-LNPs treated animals to see whether we could measure enhanced knockdown in the liposome group compared to the control group 24 h after treatment. There was no knockdown of AR mRNA in the control or the liposome groups (Fig. S5). We reasoned that this could be due to the fact that PEG-DSG-LNPs are known to remain in circulation for longer than PEG-DMG-LNPs, potentially reaching the tumor more but at the cost that PEG-DSG reduces the functional delivery of mRNA.

### 3.6 Liposomes did not reduce LNP uptake in macrophage- and hepatocyte-derived cell lines

Given that these results were not what we expected from our interpretation of previously reported data (Saunders et al., 2020), we decided to run a final *in vitro* test with the tested liposomes to verify if pre-treatment of mouse hepatocyte and macrophage immortalized cell lines would lead to reduced uptake of our siRNA-LNPs. For that purpose, we decided to include -next to our 2 LNP formulations- a third LNP formulation (cKK-E12-DMG LNPs) that was used in a previous study, where liposomes were effectively employed *in vivo* to reduce LNP clearance by the RES (Saunders et al., 2020).

**Fig. 4:**
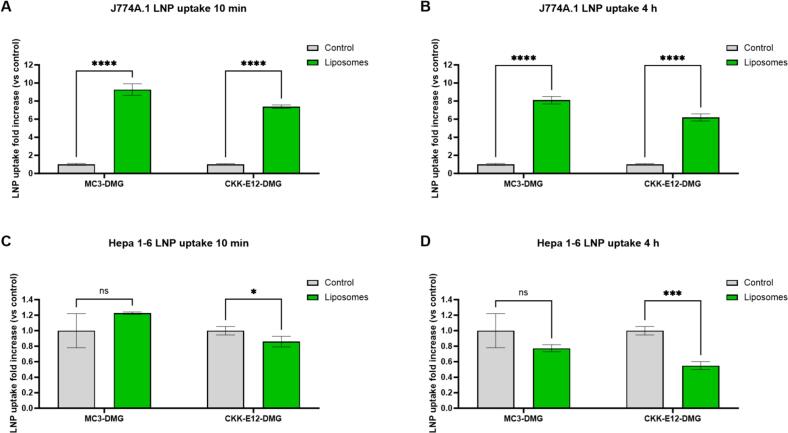
Liposome pre-treatment does not block siRNA-LNP uptake in hepatocyte and macrophage immortalized mouse cell lines. Liposomes were added to J774A.1 (A,B) and Hepa 1–6 cells (C,D) at a lipid concentration of 0.5 mg/ml for 10 min (A,C) or 4 h (B,D) in full medium. Following removal of the medium, Cy5-LNPs were added at a concentration of 100 ng encapsulated siRNA per well. The cells were then incubated for 2 h, washed, detached and LNP uptake was analyzed by flow cytometry (Cy5 MFI). A two-tailed unpaired *t*-test was performed comparing the mean fluorescent intensity (MFI) fold increase normalized to the control group. ****, *p*-value <0.0001; ***, *p*-value <0.001; **, *p*-value <0.01; *, *p*-value <0.05; ns: no significant difference. Data represent mean ± SD (*n* = 3 wells) with at least 5000 cells per well.

From our *in vitro* experiments conducted with immortalized murine macrophages (J774A.1), it became evident that pre-treatment with liposomes for 10 min (Fig. 4a) or 4 h (Fig. 4b) not only failed to decrease LNP uptake but actually led to enhanced LNP uptake for both tested formulations. When we assessed uptake in immortalized murine hepatocytes (Hepa 1–6), pre-treatment with liposomes for 10 min (Fig. 4c) did not show major differences when compared to the control group for MC3-DMG and cKK-E12-DMG LNPs. However, pre-treatment of Hepa 1–6 with liposomes for 4 h (Fig. 4d) led to a mild 23 % reduction in MC3-DMG LNP uptake and a 45 % reduction for cKK-E12-DMG LNPs.

In addition, we tested the effects of liposomes on MC3-DSG LNP uptake (Fig. S6). The MFI values were close to background signal, as the cellular uptake of PEG-DSG LNPs is limited for such short period of time (2 h). Nevertheless, pre-treatment during 10 min (Fig. S6a,c) and 4 h (Fig. S6b,d) with liposomes resulted in increased macrophage and hepatocyte uptake of MC3-DSG LNPs.

Altogether, these data underline that liposome and dextran sulfate pre-treatments did not improve siRNA-LNP circulation kinetics or tumor accumulation *in vivo*, raising questions about the effectiveness of RES blockade strategies for enhanced tumor delivery.

## 4 Discussion

The results of our study provide valuable insights into the potential of liposomes and dextran sulfate pre-treatment strategies to enhance pharmacokinetics, biodistribution, and tumor accumulation of siRNA-LNPs. Here, we discuss the implications of our findings and their significance in the context of systemic siRNA-LNP delivery for solid tumor therapy.

Our study initially set out to validate the gene silencing potential of siRNA-LNPs by silencing the AR gene in PCa. The observed reductions in AR mRNA and protein levels in two PCa cell lines (Fig. 1) confirm the feasibility and therapeutic relevance of siRNA-LNPs for modulating the AR pathway, which is a key driver of prostate cancer progression (Heinlein and Chang, 2004). However, the challenge of translating such *in vitro* results into *in vivo* systems remains a critical hurdle in the development of effective nanoparticle-based therapies.

Moving to our *in vivo* studies, we expected that pre-treatment with liposomes and dextran sulfate would reduce RES-nanoparticle clearance and therefore enhance the circulation time and tumor accumulation of DSG-siRNA-LNPs. However, contrary to these expectations, neither pre-treatment improved the circulation time or tumor uptake of DSG-siRNA-LNPs in our xenograft mouse model (Fig. 2). These findings questioned the effectiveness of the attempted RES blockade strategies for enhancing LNP tumor delivery.

Based on the obtained results for LNP circulation times (Fig. 2b), we reasoned that short-circulating LNPs might be more affected by RES blockade pre-treatment than long-circulating ones, as the RES may not be effectively continuously blocked for the entire prolonged clearance time of the latter. As pre-treatment with dextran sulfate exhibited clear signs of toxicity in treated animals, we decided to exclude this treatment from this follow-up study. However, even when testing this hypothesis using PEG-DMG (short-circulating) and PEG-DSG (long-circulating) siRNA-LNPs in a PCa xenograft model, pre-treatment with liposomes did not lead to a significant improvement in LNP circulation times or tumor accumulation (Fig. 3).

Based on the obtained results, we theorize that our LNPs and the liposomes might be cleared by different (sub)populations of RES cells, probably due to varying nanoparticle protein corona profiles (Walkey et al., 2012). The term “nanoparticle protein corona” refers to the layer of proteins that adsorb onto the surface of nanoparticles when they are present in biological environments, such as blood or tissue (Mahmoudi et al., 2023). This corona significantly influences the nanoparticles' biological behavior, affecting their circulation time, tissue distribution, cellular uptake, and overall efficacy in drug delivery applications (Monopoli et al., 2012; Lynch et al., 2006; Walczyk et al., 2010). Consequently, different nanoparticle protein corona profiles between the liposomes and the LNPs could explain why pre-treatment with liposomes did not lead to enhanced circulation time or tumor accumulation of our LNPs. These claims are in line with suggestions made in other publications regarding Kupffer cells constituting a diverse population, with each cell specialized in the clearance of a particular type of nanoparticle (Ouyang et al., 2020). Another possible explanation could be that MC3-LNPs are taken up by hepatocytes through apoE-mediated uptake, which could compensate for the RES blockade.

Another possible explanation for the lack of efficiency of the liposomes may have lied in the fact that in our studies each animal received ∼9 × 10^12^ DMG-LNPs or DSG-LNPs, suggesting that we dosed above the recently suggested 10^12^ particles threshold for enhanced tumor accumulation (Ouyang et al., 2020). We speculate that the above-threshold LNP dose used in these experiments might have masked the potential effects of RES blockade, as the high LNP dose itself already might lead to enhanced circulation times and tumor accumulation of the particles. This hypothesis reinforces the earlier explanation regarding the varying protein corona profiles of different nanoparticles. It suggests that when injecting above the 10^12^ nanoparticle threshold, the particles with the same protein corona may be blocking the RES clearance of surplus particles, thereby enhancing their tumor accumulation.

In an attempt to gain a deeper understanding of our *in vivo* results, we also conducted *in vitro* experiments using mouse hepatocyte and macrophage cell lines to examine how liposomes affect the cellular uptake of siRNA-LNPs. Unexpectedly, pre-treatment with the *in vivo* tested liposomes led to enhanced macrophage LNP uptake (Fig. 4a,b). We hypothesize that this increased uptake might result from the fact that preincubation with liposomal particles activates the phagocytic capacity of macrophages, thereby enhancing LNP uptake. Previous reports have described how liposomal cholesterol can act as an enhancer of phagocytic capacity of macrophages (Ghosh et al., 2014). Interestingly, LNP uptake in hepatocyte-derived cells was not clearly reduced after liposome pre-treatment, particularly for MC3-LNPs. These results could explain why we did not observe a decrease in LNP liver accumulation or enhanced circulation time and tumor accumulation when pre-treating the animals with liposomes in our *in vivo* experiments. Alternatively, the liposomes present in circulation at the time of LNP administration may contribute to LNP destabilization, thereby reducing their circulation time.

Moreover, in this study we used an immunodeficient strain of mice to engraft human tumors in an attempt to provide better predictions of the effects of our LNPs on these tumors. Employing immunodeficient mice may be justified for studying the effects of low molecular weight drugs in human tumors. However, it is crucial to consider that with more complex systems like nanomedicines, the possibility of missing critical interactions with the immune system could outweigh the advantages of using human tumor models compared to syngeneic tumors (Jones et al., 2013; La-Beck and Gabizon, 2017; Shmeeda et al., 2016; Rios-Doria et al., 2015).

The ineffectiveness of the liposomes and dextran sulfate pre-treatments to improve siRNA-LNP pharmacokinetics suggests that these pre-treatments are not universally beneficial in preventing nanoparticle clearance. Future research could shift focus from RES blockade strategies to developing long-circulating particles, such as PEG-DSG LNPs, that retain the transfection efficiency of short-circulating particles like PEG-DMG LNPs. Additionally, future studies could evaluate which liver cell types are reached by the liposomes compared to the LNPs. Further research is needed to better understand the molecular mechanisms involved in LNP clearance and to develop tailored pre-treatment strategies that specifically block key receptors, potentially leading to a more favorable pharmacokinetic profile and tumor accumulation. Implementing RES transient blockade could enhance LNP-based cancer therapies, aligning with precision medicine goals to maximize tumor efficacy and minimize toxicity.

## Declaration of generative AI and AI-assisted technologies in the writing process

During the preparation of this work the author(s) used ChatGPT - OpenAI in order to improve the fluency of the written text. After using this tool/service, the author(s) reviewed and edited the content as needed and take(s) full responsibility for the content of the publication.

## CRediT authorship contribution statement

**Pol Escudé Martinez de Castilla:** Writing – review & editing, Writing – original draft, Visualization, Validation, Methodology, Investigation, Formal analysis, Data curation, Conceptualization. **Mariona Estapé Sentí:** Investigation. **Sigrun Erkens:** Methodology. **Wytske M. van Weerden:** Resources. **Sander A.A. Kooijmans:** Methodology, Investigation. **Marcel H. Fens:** Writing – review & editing, Validation, Methodology, Investigation. **Pieter Vader:** Writing – review & editing, Validation, Supervision, Methodology, Formal analysis, Data curation, Conceptualization. **Raymond M. Schiffelers:** Writing – review & editing, Validation, Supervision, Resources, Project administration, Methodology, Funding acquisition, Formal analysis, Data curation, Conceptualization.

## Declaration of competing interest

The authors declare the following financial interests/personal relationships which may be considered as potential competing interests:

Raymond Schiffelers reports financial support was provided by European Commission Marie Sklodowska-Curie Actions. Raymond Schiffelers reports financial support was provided by H2020 Health. Raymond Schiffelers reports a relationship with Nanocell Therapeutics that includes: board membership and employment. If there are other authors, they declare that they have no known competing financial interests or personal relationships that could have appeared to influence the work reported in this paper.

## Data Availability

Data will be made available on request.

## Appendix A Supplementary data

Supplementary material RES blockade. Image 1
