# Supplementary material for: Reticuloendothelial system blockade does not enhance siRNA-LNP circulation or tumor accumulation in mice
Source: Int J Pharm X. 2025 Feb 27;9:100324. doi: 10.1016/j.ijpx.2025.100324 (PMC11925117; doi:10.1016/j.ijpx.2025.100324)
Supplement: Supplementary file 1 — Supplementary material RES blockade. [file mmc1.docx]

**Supplementary information**

**Table S1 | Nanoparticle characterization details from all experiments.** Size, PdI, Zeta potential and RNA encapsulation efficiency were measured for all the tested nanoparticles.

| **Experiment** | **Nanoparticles** | **Size**  **(d. nm)** | **PdI**  **(0-1)** | **Zeta potential (mV)** | **RNA encapsulation efficiency (%)** |
| --- | --- | --- | --- | --- | --- |
| *In vitro* AR knockdown | DMG-siNT-LNPs | 68 | 0.17 | -4.8 | 99 |
| *In vitro* AR knockdown | DMG-siAR-LNPs | 70 | 0.26 | -4.3 | 99 |
| *In vivo* PC346C biodistribution | DSG-siAR-LNPs | 72 | 0.12 | -2.2 | 97 |
| *In vivo* PC346C  and LNCaP biodistribution & *In vitro*  liposomes  pre-treatment | Liposomes | 296 | 0.20 | -73.6 |  |
| *In vivo* LNCaP biodistribution | DMG-siAR-LNPs | 65 | 0.07 | 0.8 | 99 |
| *In vivo* LNCaP biodistribution | DSG-siAR-LNPs | 69 | 0.06 | 0.1 | 98 |
| *In vitro*  liposomes  pre-treatment | DMG-siAR-LNPs | 67 | 0.15 | 1.0 | 99 |
| *In vitro*  liposomes  pre-treatment | DSG-siAR-LNPs | 69 | 0.15 | 0.2 | 99 |
| *In vitro*  liposomes  pre-treatment | cKK-E12-LNPs | 63 | 0.44 | -1.5 | 97 |


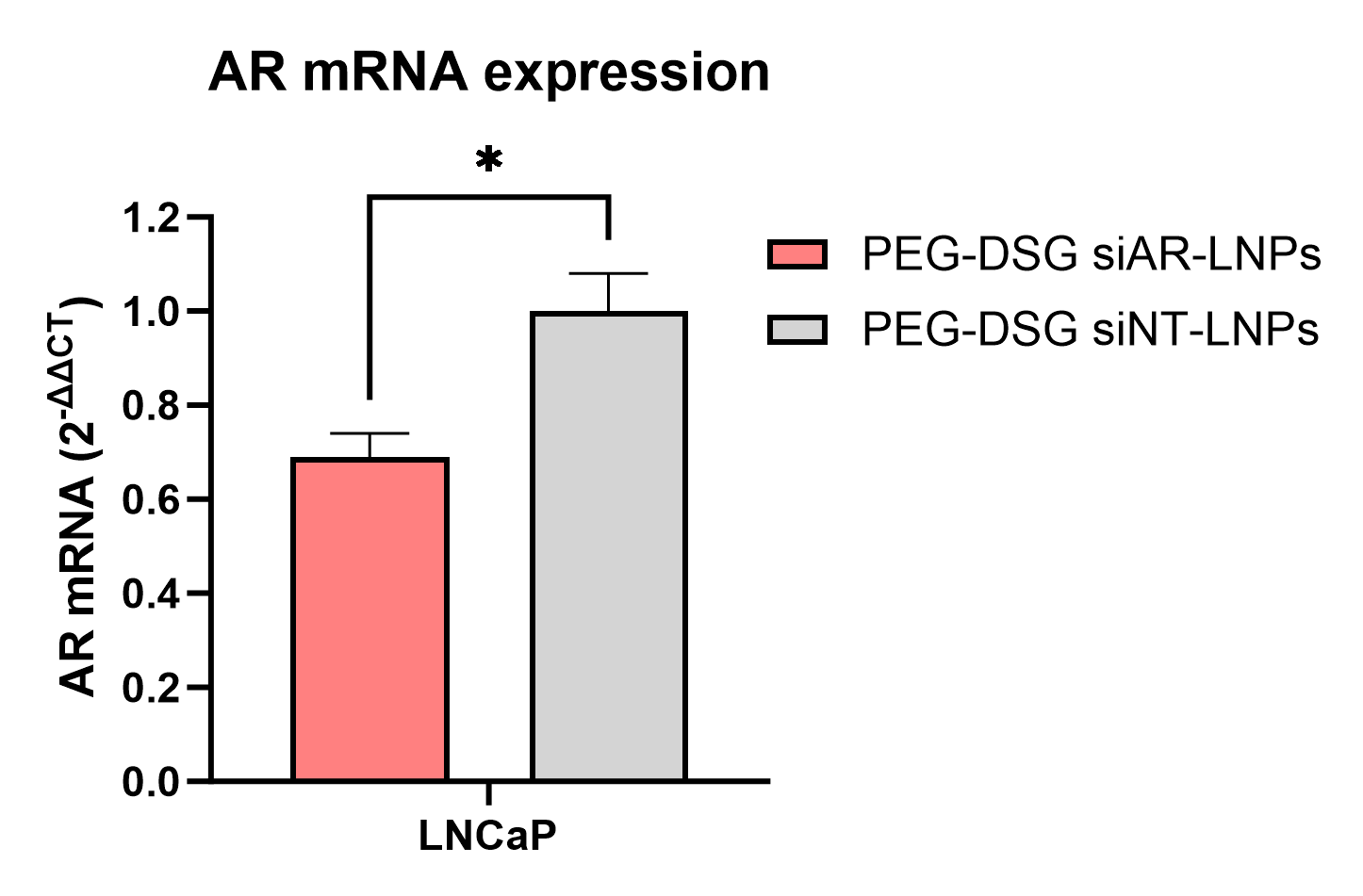


**Figure S1 |** **PEG-DSG siAR-LNPs knock down AR *in vitro* in LNCaP as demonstrated by RTqPCR.** PEG-DSG siRNA-LNPs (0.5 µg/ml per well) were added to 12-well plates. AR mRNA expression was assessed using RT-qPCR and normalized to GAPDH 24 hours after LNP addition. Data represent mean ± SD (n=3 biological replicates), and significance of the differences between groups was determined by a two-tailed unpaired t-test. *, *p*-value <0.05.





**Figure S2 | LNPs and liposomes are stable over the course of 60 days at 4 °C.** Variations in PEG-DMG LNPs, PEG-DSG LNPs and liposomes size **(A)**, polydispersity index (PdI) **(B)**, Z-potential **(C)**, and RNA encapsulation efficiency **(D)** over time were assessed using a two-way ANOVA with Šidák correction for multiple comparisons. No statistically significant differences were found. A,B were measured by dynamic light scattering (DLS), C was measured using a Zetasizer and D using a Quant-It™ Ribogreen RNA Assay kit. The results are represented by mean ± SD (n=3 measurements).





**Figure S3 | LNPs are stable when mixed with dextran sulfate in PBS at room temperature.** Assuming a mouse weighing 30 grams would have a total blood volume (TBV) of approximately 1.75 mL, we mixed siAR-LNPs corresponding to 5 mg siRNA/kg of mouse (150 µg) with dextran sulfate corresponding to 30 mg/kg (0.9 mg) in 1.75 mL PBS pH 7.4 for 2 h at room temperature. We then measured LNP size **(A)** and LNP polydispersity index (PdI) **(B)** by dynamic light scattering (DLS). Unpaired two-tailed t-tests were employed for group comparisons and no significant differences were observed. DLS data represented by mean ± SD (n=3 measurements).

**A**

**PEG-DMG LNPs Cy5.5 siRNA**

**Liposomes + PEG-DMG LNPs Cy5.5 siRNA**




**B**

**PEG-DMG LNPs DSPE-Cy7**

**Liposomes + PEG-DMG LNPs DSPE-Cy7**




**C**

**PEG-DSG LNPs Cy5.5 siRNA**

**Liposomes + PEG-DSG LNPs Cy5.5 siRNA**




**D**

**PEG-DSG LNPs DSPE-Cy7**

**Liposomes + PEG-DSG LNPs DSPE-Cy7**







**F**

**E**

**PBS (Cy5.5 scale) PBS (Cy7 scale)**




**Figure S4 |** **Whole-organ fluorescence spectroscopy for Cy5.5 and Cy7 fluorescence in LNCaP-engrafted mice treated with PEG-DMG or PEG-DSG siRNA-LNPs, revealed no major differences between liposome and control groups.** NMRI-nu immunodeficient mice bearing LNCaP tumors were systemically injected with liposomes (360 mg/kg) or PBS, followed by i.v. injection of dually labeled (Cy5.5-siRNA and Cy7-DSPE) PEG-DMG **(A,B)** or PEG-DSG **(C,D)** siAR-LNPs at a dose of 2.5 mg/kg siRNA or PBS (- control) **(E,F)**. 6 h after PEG-DMG-LNPs and 24 h after PEG-DSG-LNPs administration mice were sacrificed, organs perfused with PBS and total organ fluorescence was measured on a Pearl Impulse Imager. Pictures represent n=4-5 animals for LNP treated animals and n=2 for PBS treated animals.

**
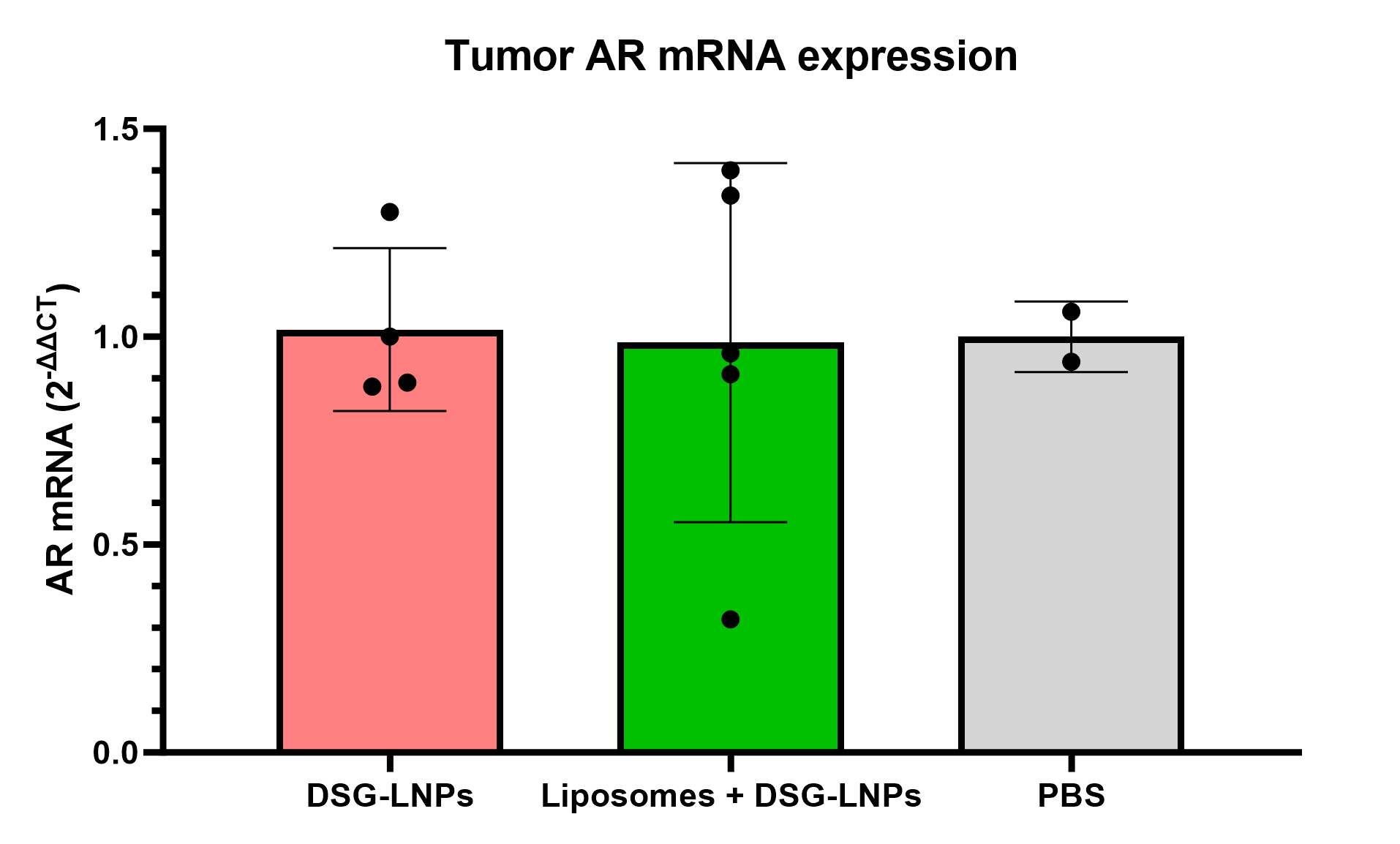
**

**Figure S5 |** **After treatment with DSG-LNPs or liposomes + DSG-LNPs, no AR mRNA knockdown was detected in LNCaP tumors 24 h post-treatment.** The AR mRNA expression was assessed by RTqPCR in tumor tissue lysates and normalized to GADPH mRNA expression. The intravenous administration of liposomes (360 mg/kg) and 2.5 mg/kg siRNA per mouse occurred 10 minutes apart. A One-Way ANOVA with Tukey’s multiple comparisons test was used to compare the mean signals between the treatment groups and the control group, with n=4-5 for both DSG-LNPs and liposome groups, and n=2 for the PBS group. No significant differences were observed between groups.


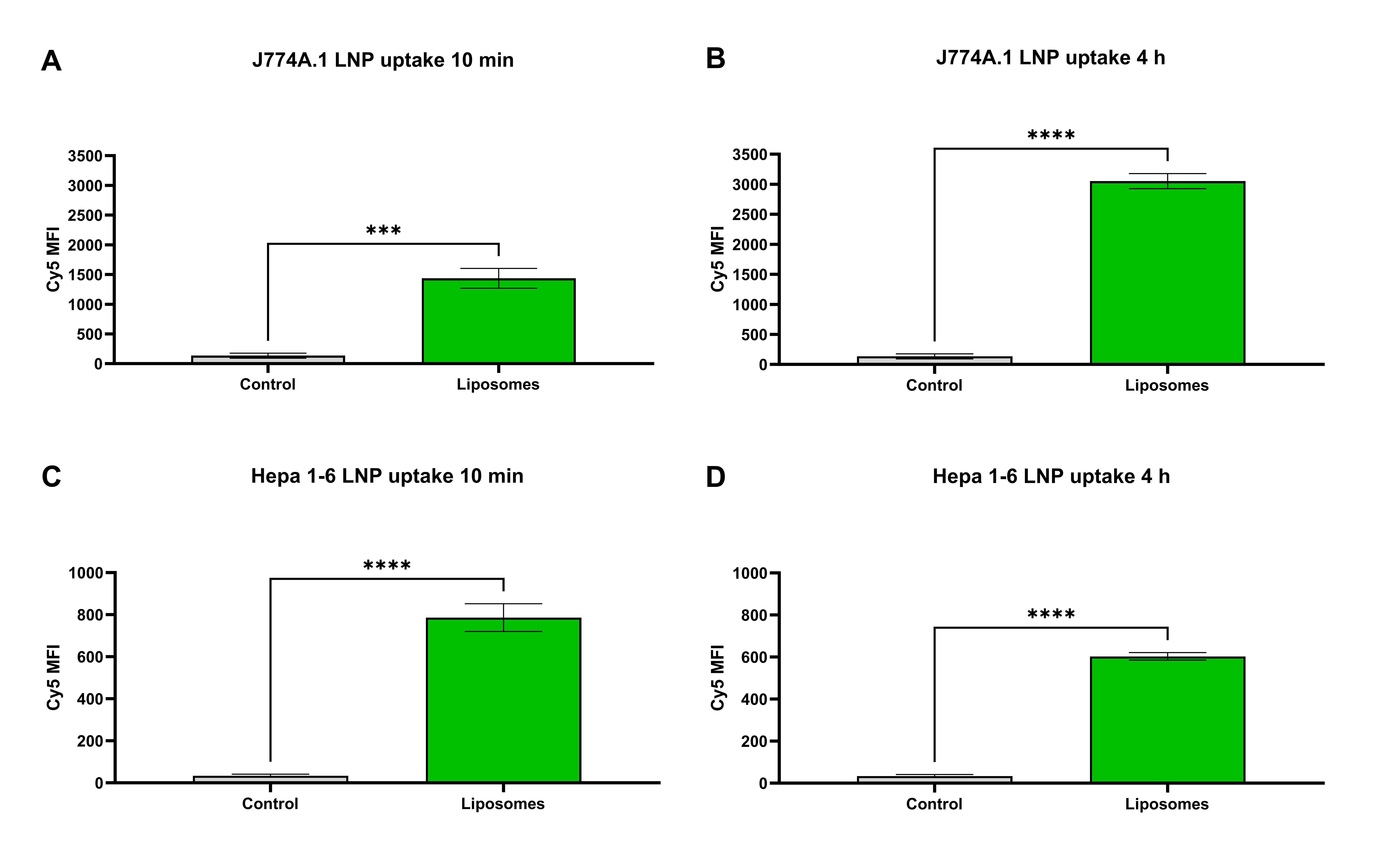


**Figure S6 | Pre-treatment with liposomes enhanced MC3-DSG-LNP uptake in macrophage and hepatocyte immortalized mouse cell lines.** Liposomes were added to J774A.1 **(A,B)** and Hepa 1-6 cells **(C,D)** at a lipid concentration of 0.5 mg/ml for 10 minutes (A,C) or 4 h (B,D) in full medium. Following removal of the medium, Cy5-LNPs were added at a concentration of 100 ng encapsulated siRNA per well. The cells were then incubated for 2 h, washed, detached and LNP uptake was analyzed by flow cytometry (Cy5 MFI). A two-tailed unpaired t-test was performed comparing the mean uptake signals between the treatment groups and the control group. ****, *p*-value <0.0001; ***, *p*-value < 0.001. Data represent mean ± SD (n=3 wells) with at least 5000 cells per well.
